# Impact of large language model (ChatGPT) in healthcare: an umbrella review and evidence synthesis

**DOI:** 10.1186/s12929-025-01131-z

**Published:** 2025-05-07

**Authors:** Usman Iqbal, Afifa Tanweer, Annisa Ristya Rahmanti, David Greenfield, Leon Tsung-Ju Lee, Yu-Chuan Jack Li

**Affiliations:** 1 Institute for Evidence-Based Healthcare, Faculty of Health Sciences & Medicine, Bond University, Gold Coast, Australia; 2 Evidence-Based Practice Professorial Unit, Gold Coast Hospital & Health Service (GCHHS), Gold Coast, QLD Australia; 3 Department of Nutrition & Dietetics, School of Health Sciences, University of Management and Technology, Lahore, Pakistan; 4 Department of Health Policy and Management, Faculty of Medicine, Public Health and Nursing, Universitas Gadjah Mada, Yogyakarta, Indonesia; 5 Department of Computer Science, Faculty of Science and Technology, Middlesex University, London, UK; 6 School of Population Health, Faculty of Medicine and Health, University of New South Wales (UNSW), Sydney, Australia; 7 Graduate Institute of Clinical Medicine, Taipei Medical University, Taipei, Taiwan; 8 Department of Dermatology, Taipei Medical University Hospital, Taipei Medical University, Taipei, Taiwan; 9 Department of Dermatology, School of Medicine, College of Medicine, Taipei Medical University, Taipei, Taiwan; 10 Graduate Institute of Biomedical Informatics, College of Medical Science and Technology, Taipei Medical University, Taipei, Taiwan; 11 Department of Dermatology, Taipei Municipal Wanfang Hospital, Taipei Medical University, Taipei, Taiwan; 12 International Center for Health Information Technology, Taipei Medical University, Taipei, Taiwan

**Keywords:** ChatGPT; Large language models (LLMs), Generative AI, Healthcare; Medical research; Medical education; Patient care, Consumer health, Reviews, Evidence synthesis

## Abstract

**Background:**

The emergence of Artificial Intelligence (AI), particularly Chat Generative Pre-Trained Transformer (ChatGPT), a Large Language Model (LLM), in healthcare promises to reshape patient care, clinical decision-making, and medical education. This review aims to synthesise research findings to consolidate the implications of ChatGPT integration in healthcare and identify research gaps.

**Main body:**

The umbrella review was conducted following Preferred Reporting Items for Systematic Reviews and Meta-Analyses (PRISMA) guidelines. The Cochrane Library, PubMed, Scopus, Web of Science, and Google Scholar were searched from inception until February 2024. Due to the heterogeneity of the included studies, no quantitative analysis was performed. Instead, information was extracted, summarised, synthesised, and presented in a narrative form. Two reviewers undertook title, abstract, and full text screening independently. The methodological quality and overall rating of the included reviews were assessed using the A Measurement Tool to Assess systematic Reviews (AMSTAR-2) checklist. The review examined 17 studies, comprising 15 systematic reviews and 2 meta-analyses, on ChatGPT in healthcare, revealing diverse focuses. The AMSTAR-2 assessment identified 5 moderate and 12 low-quality reviews, with deficiencies like study design justification and funding source reporting. The most reported theme that emerged was ChatGPT's use in disease diagnosis or clinical decision-making. While 82.4% of studies focused on its general usage, 17.6% explored unique topics like its role in medical examinations and conducting systematic reviews. Among these, 52.9% targeted general healthcare, with 41.2% focusing on specific domains like radiology, neurosurgery, gastroenterology, public health dentistry, and ophthalmology. ChatGPT’s use for manuscript review or writing was mentioned in 17.6% of reviews. Promising applications include enhancing patient care and clinical decision-making, though ethical, legal, and accuracy concerns require cautious integration.

**Conclusion:**

We summarise the identified areas in reviews regarding ChatGPT's transformative impact in healthcare, highlighting patient care, decision-making, and medical education. Emphasising the importance of ethical regulations and the involvement of policymakers, we urge further investigation to ensure the reliability of ChatGPT and to promote trust in healthcare and research.

**Supplementary Information:**

The online version contains supplementary material available at 10.1186/s12929-025-01131-z.

## Background

Recent advancements in Artificial Intelligence (AI) have brought transformative changes across various industries, including healthcare [16]. AI-powered tools and technologies offer the potential to revolutionise healthcare delivery, improving patient outcomes, and enhancing clinical decision-making processes. Among these technologies, ChatGPT (Chat Generative Pre-trained Transformer), a Large Language Model (LLM), developed by OpenAI, has received significant attention within the healthcare sector [12]. As a state-of-the-art Natural Language Processing (NLP) model trained on a vast corpus of text data, ChatGPT can generate human-like responses to text inputs. Operating on deep learning principles and employing a transformer architecture, ChatGPT surpasses traditional rule-based chatbots by not relying on predefined rules or templates for generating responses [23]. Instead, it leverages its extensive pre-trained knowledge to understand and respond to queries in a contextually relevant manner.

ChatGPT is a promising tool for a wide range of diverse and multifaceted applications in consumer health [23]. One of the primary areas of promise is in healthcare education. With its ability to generate informative and educational content, ChatGPT can serve as a valuable resource for medical students, healthcare professionals, and educators [25]. It can assist in the creation of educational materials, answer clinical queries, and facilitate interactive learning experiences. Additionally, ChatGPT can aid medical research by generating human-like text, offering fundamental guidance, and elucidating complex concepts [1]. In clinical practice, ChatGPT has the potential to streamline clinical documentation, patient communication, and decision support tasks, thus improving the workflow efficiency. By automating routine administrative tasks and providing real-time assistance, ChatGPT can help reduce the burden on healthcare professionals and enhance the quality of patient care [14]. Moreover, ChatGPT holds promise in diagnostic assistance and decision support. Its ability to process and analyze medical data, including patient histories, symptoms, and diagnostic tests, enables it to provide valuable insights and recommendations to healthcare providers. In fields such as radiology and pathology, ChatGPT can assist in image interpretation, differential diagnosis, and treatment planning, potentially improving diagnostic accuracy and patient outcomes [21]. Furthermore, it can offer second opinions on dermatological treatments, which may become increasingly reliable as it continues to optimize [33].

Despite its potential benefits, integrating ChatGPT into healthcare practice poses risks, challenges and limitations. Addressing risks associated with ethical concerns regarding patient privacy, data security, and algorithmic bias is crucial for ensuring its safe and responsible use. Moreover, verifying the accuracy, reliability, and trustworthiness of ChatGPT-generated content requires further investigation [34].

Translating promises into reality is always a significant step. The potential uses and benefits of ChatGPT for consumer health have emerged but are not yet fully realised. Further work is necessary to understand for what and how ChatGPT is being used. Similarly, understanding the risks, challenges and limitations of ChatGPT in consumer health can help ensure its appropriate and effective use. Given these considerations, conducting an umbrella review of systematic reviews on ChatGPT in consumer health is imperative. This study aims to provide a comprehensive overview through synthesis and evaluation, including evidence gap synthesis, discerns implications for integration, and highlight areas for further research and development.

## Methods

An umbrella review synthesises existing systematic reviews and meta-analyses on a topic, offering a comprehensive overview of evidence from multiple studies. It provides a broader perspective, enhancing research depth and reliability. Thus, it is ideal for exploring ChatGPT’s applications and impacts in healthcare. This umbrella review was conducted according to the Preferred Reporting Items for Systematic Reviews and Meta-Analysis Protocols guidelines (PRISMA) [26]. The checklist recommended by Choi et al. [8] was followed for conducting and reporting this umbrella review. The protocol was registered with the International Prospective Register of Systematic Reviews (PROSPERO) under registration number CRD42024510926.

### Search strategy and eligibility criteria

The search strategy for included reviews was conducted across five electronic databases: (i) Cochrane Library (the Cochrane Database of Systematic Reviews); (ii) PubMed; (iii) Web of Science (all databases); (iv) Scopus; and (v) Google Scholar. The review period was up until February 3, 2024, without restrictions on language or publication year.

To capture a wide-ranging collection of reviews covering ChatGPT’s role in consumer health, we performed a comprehensive searching strategy across the database by using a combination of keywords and Boolean operators. For Google Scholar, specific filters for 'review articles' and sorting by relevance were applied, followed by a targeted search query to refine the results further. The detailed search strategy can be found in Additional file 1.

The inclusion criteria were: (i) peer-reviewed Systematic Reviews (SR) and Systematic Reviews with Meta-Analysis (SRMA); (ii) focusing on ChatGPT within the scope of consumer health, specifically highlighting areas such as patient education, health information seeking, digital health interventions, health literacy, and various forms of electronic health services. While prioritising SR and SRMA, the scope extends to any study following PRISMA guidelines, thereby ensuring a broad yet rigorous collection of literature on ChatGPT's impact on consumer health informatics. Studies were excluded if they: (i) were not SR or SRMA; (ii) lacked relevance to ChatGPT in consumer health; (iii) were not entirely in English; or (iv) were only available as abstracts without full texts.

For study selection, two reviewers (AT and ARR) independently performed the literature search across the selected databases, then screened titles and abstracts to eliminate duplicates, and read the full texts of all papers to identify relevant systematic reviews. Any disagreements were resolved by a consensus with a third reviewer (UI).

### Assessment of methodological quality

The methodological quality of the included reviews was appraised using A MeaSurement Tool to Assess systematic Reviews (AMSTAR-2) guideline, a comprehensive framework to determine the thoroughness and reliability of the reviews [28]. Quality levels were categorised into high, moderate, low, or critically low, based on the presence of critical flaws and non-critical weaknesses. The AMSTAR-2 appraisal was initially performed by a single reviewer (ARR) and then verified by another (AT), with both agreeing on the evaluation outcomes without any disagreement.

### Data extraction and evidence synthesis

The selected articles were manually reviewed, and pertinent information was extracted, synthesised, and summarised in tabular format. We did not perform quantitative analysis in this review given the heterogeneity of included articles and because the meta-analysis had not been performed in most of the articles. The findings were synthesised into main and sub-themes, analysing the most common outcomes and methodological quality of the systematic reviews.

## Results

**Fig. 1:**
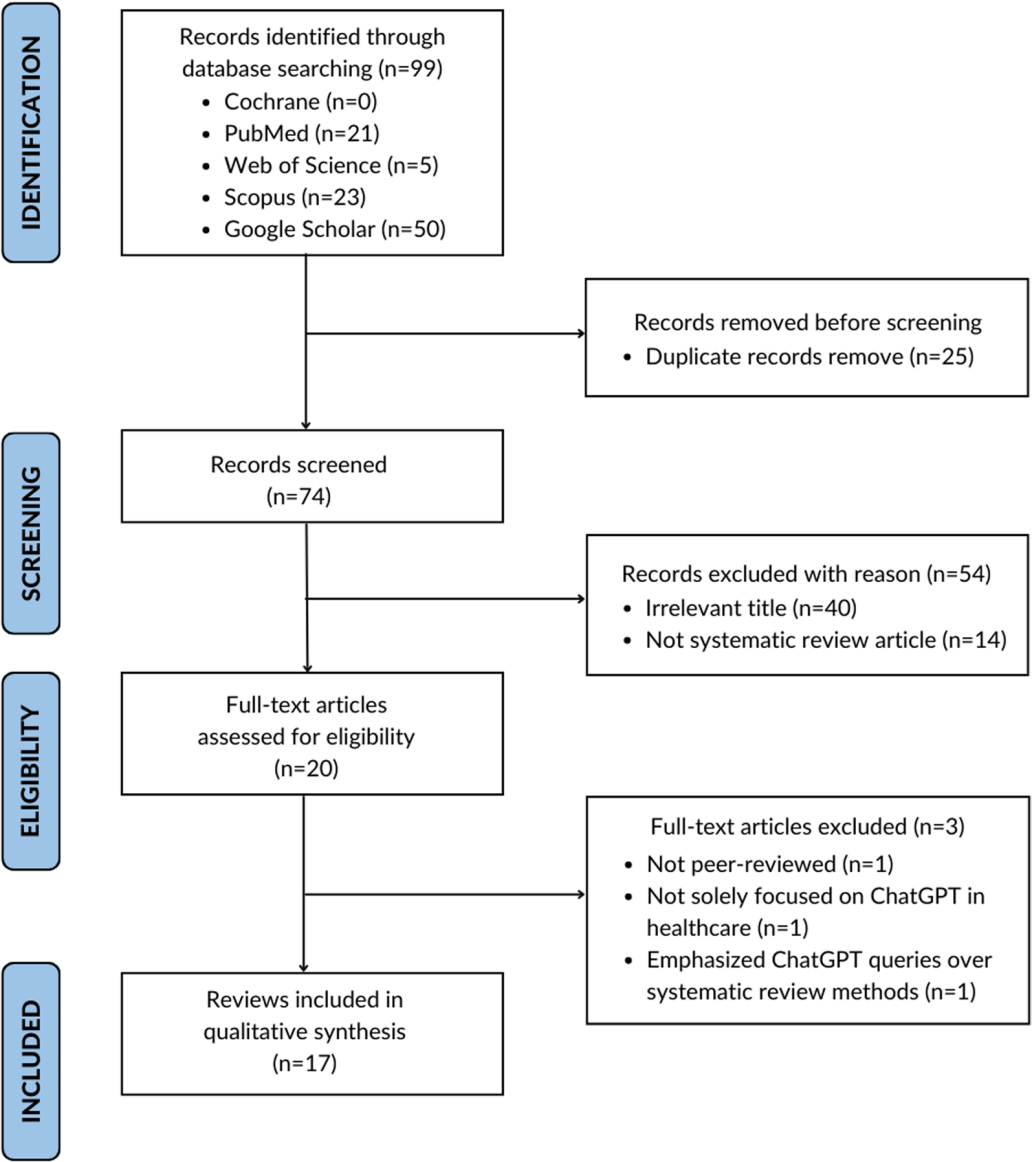
PRISMA flow diagram representing the inclusion of systematic reviews and meta-analysis

A preliminary search with the key term “ChatGPT” yielded no results in the Cochrane Library. Subsequently, a search was conducted on PubMed using keyword “ChatGPT” with the filter set to “Systematic Reviews” without any restriction for date, yielding 21 records. In addition, after applying the filter of “review articles” and “sort by relevance”, we inserted a predefined search query in the search tab of Google Scholar, producing 433 results. As Google Scholar gives very broad search results, we utilised the methodology described by Haddaway et al. [11] and included the first 50 records for further screening. Out of these, 15 titles were deemed relevant to the objectives of this umbrella review. Searches in Scopus and Web of Science, following predefined criteria, yielded 23 and 5 records, respectively. After removing the duplicates, 74 unique titles were screened by titles and abstracts, leading to the exclusion of 54 studies for being irrelevant to the study objective (40) or not being systematic reviews (14), out of which 20 were deemed eligible for full-text analysis. Three articles were further excluded during full-text study for not solely focusing on ChatGPT [19], being a preprint (not peer-reviewed) [9], and primarily emphasising practical query interactions with ChatGPT, rather than providing a comprehensive analysis of systematic review results [7]. Therefore, 17 articles were included in the final group for analysis and synthesis, as illustrated in the PRISMA flow diagram (Fig. 1). Of these, 15 comprised systematic reviews, with an additional 2 being meta-analyses. The distribution of publication years underscores the topic's emerging relevance, with 13 articles published in 2023 and 4 in 2024 to February, indicating a notable surge in ChatGPT-related research during this period.

The methodological quality assessment using AMSTAR-2, as summarised in Table 1, indicated that 5 out of 17 reviews (29.4%) were of moderate quality, while the remaining 12 out of 17 reviews (70.6%) had low quality. Notably, the most frequently lacking quality indicator was the explanation or justification of the selection of study designs for inclusion in the review (item number 3). Furthermore, deficiencies were observed in reporting the sources of funding (item number 10), addressing the risk of bias when discussing results (item number 13), and explaining or discussing observed heterogeneity (item number 14), indicating areas needing improvement in future reviews.

**Table 1:**
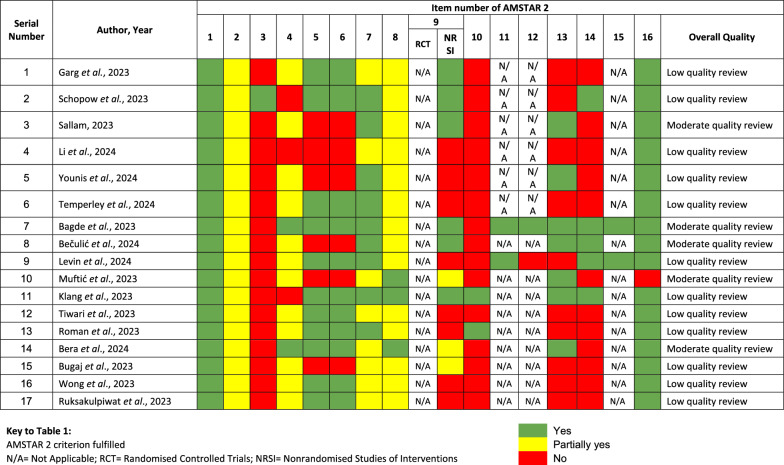
Risk of Bias analysis of included systematic reviews using AMSTAR-2 [28]

The AMSTAR-2 guideline assesses 16 criteria, including: (i) research questions and inclusion criteria based on the PICO framework; (ii) a pre-defined methodological protocol; (iii) the rationale for including specific study designs; (iv) a comprehensive literature search strategy; (v) duplication in study selection; (vi) duplication in data extraction; (vii) justification for excluded studies; (viii) a detailed description of included studies; (ix) risk of bias assessment; (x) funding source reporting for included studies; (xi) use of appropriate meta-analysis statistical methods; (xii) assessment of risk of bias impact on results; (xiii) consideration of risk of bias in outcomes interpretation; (xiv) a satisfactory explanation on observed heterogeneity; (xv) an adequate investigation of publication bias; and (xvi) reporting of potential sources of conflict of interest

Tables 2 and 3 present a summary of the methodology aims and key findings of the included articles, offering a comprehensive overview of how ChatGPT is being integrated and evaluated within healthcare settings. These reviews exhibited considerable heterogeneity in terms of their fields and objectives. The majority (82.35% or 14 out of 17) of the reviews focused on elucidating the usage, advantages, and limitations of ChatGPT across various domains within healthcare. The remaining three reviews explored unique topics, including the role of ChatGPT in multiple-choice question-based medical examinations [17], medical research [24], and performance in systematic review tasks [27]. While a majority (52.94% or 9 out of 17) of the reviews concentrated on general healthcare practices, a total of seven reviews delved into specific domains such as radiology [4, 31], neurosurgery [3],Roman, Al-Sharif, & Gharyani, 2023), gastroenterology [15], public health dentistry [32] and ophthalmology [35]. Quantitative analysis of pooled findings was conducted in two studies. It was noted that 3 out of the 17 systematic reviews (17.65%) incorporated ChatGPT to aid in reviewing [10, 27] or writing manuscripts [20], acknowledging the contribution in their acknowledgements section.

**Table 2:** Summary of methodologies of included systematic reviews and systematic reviews with meta-analysis (n = 17)

Author, year	Country (corresponding author’s affiliation)	Search Strategy (databases, search terms and timeline)	RoB* assessment performed (Y/N)	Number and type of studies included	Meta-analysis performed (Y/N)	Version of ChatGPT studied	Utilization of ChatGPT (in writeup/ conducting systematic search)	Acknowledgement of ChatGPT by authors	Funding information
[10]	Lucknow, India	Embase, Scopus, PubMed, Google Scholar Search term: ChatGPT Data till 24th May 2023	No	118 articles (original articles, reviews, editorial/ commentaries, and letter to the editor)	No	Not mentioned	ChatGPT was used for analysis of records and manuscript writing ChatPDF was used to generate	Yes	Authors declare no financial support
[27]	Leipzing, Germany	PubMed search MESH terms were developed by ChatGPT and refined by human authors January, 2020 onwards (NLP instead of ChatGPT only)	No	5 (main concern was to evaluate the effectiveness and reliability of ChatGPT in conducting review compared with human authors)	No	3.5 (Legacy) and 4.0	All tasks of systematic review augmented by ChatGPT	Yes	No information in the article
[25]	Amman, Jordan	PubMed/MEDLINE and Google Scholar Search term: ChatGPT Year: 2022–2023	No	60 (article, review, communication, editorial, opinion)	No	Not mentioned	No	No	Authors declare no financial support
[18]	Essen, Germany	Pubmed Search term: ChatGPT Till March, 2023	No	58 (all types but the writeup done for original articles and opinion/editorial pieces separately)	No	Version released in Nov., 2022	No	No	Medical Faculty of German Rheinisch-Westfälische Technische Hochschule Aachen University as part of the Clinician Scientist Program
[36]	Basra, Iraq	Taylor and Francis, Google Scholar, Scopus, Web of Science, Elsevier, Springer, MDPI, IEEE Xplore digital and Wiley Keywords: ChatGPT with various keywords for healthcare and medicine between November 2022 and August 2023	No	82 (All types)	No (although the title suggests so)	Not mentioned	No	No	Funding by The Deanship of Scientific Research at King Khalid University, KSA
[31]	Dublin, Ireland	PubMed, EMBASE and Web of Science Keyword not described Search till 18 June 2023	No	6 (prospective)	No	version 3.0 to 4.0	No	No	Authors declare that open access funding provided by IReL
[2]	Rajnandgaon, India	PubMed, Google Scholar, Scopus, EMBASE, Cochrane Library, and UpToDate Key terms: ChatGPT and Medical Informatics which were combined using Boolean operators “AND” and “OR” with “dental”, “specialty”, “accuracy”, “query”, “response” and “meta- analysis”. Articles published in 2023 only	Yes	11 descriptive studies	Yes	3.5	No	No	No information in the article
[3]	Zenica, Bosnia and Herzegovina	Pubmed, Embase, Scopus Keywords:(ChatGPT OR OpenAI) AND (neurosurgery OR spinal surgery) till 12th of August, 2023	No	13 (all types)	No	Not mentioned	No	No	Authors declare no financial support
[17]	Quebec, Canada	PubMed, Scopus and Web of Science Keyterm: ChatGPT till 2nd June 2023	No	19 peer-reviewed articles	yes	3.5	No	No	Authors declare no financial support
[20]	Sarajevo, Bosnia and Herzegovina	Google Scholar and PubMed Keyword: “ChatGPT applications in medicine” Till April 15, 2023	No	31- any type of published scientific research or preprints	No	Not mentioned	Yes (Paraphrasing and writeup)	Yes	No information in the article
[15]	Tel Aviv, Israel	Pubmed Key words: MESH terms related to ‘ChatGPT’, and ‘Gastroenterology’	Yes	6	No	Heterogenous	No	No	Authors declare no financial support
[32]	Rosemont, USA	Pubmed, Embase, Ovid, Global Health, PsycINFO, Web of Science Using search phrases related to chatGPT and public health dentistry Between: March 31, 2018, and March 31, 2023	No	39	No	Not mentioned	No	No	Authors declare no financial support
[22]	Abu Dhabi, UAE	PubMed, Google Scholar, and Embase MESH terms for "neurosurgery" AND "ChatGPT" Till June 30, 2023	No	22 -All types (peer reviewed and gray literature search)	No	Not specified	No	No	Authors declare no financial support
[4]	Cleveland, Ohio, USA	MEDLINE, EMBASE using the search terms: ChatGPT, imaging, radiology, LLM, large language models, BARD from November 2022 to August 15, 2023,	Yes	51 (23 original research articles and 28 non-original research articles)	No	ChatGPT version 3, 3.5 and 4	No	No	Authors declare no financial support
[5]	Bucharest, Romania	ProQuest, Scopus, and the Web of Science with search terms including "generative artificial intelligence-based diagnostic algorithms," "disease risk detection," "personalized and targeted healthcare procedures," and "patient care safety and quality." throughout April 2023	Yes	32 (28 original research articles and 4 review articles)	No	Not specified	No	No	The paper is an output of the project NFP313010BWN6 “The implementation framework and business model of the Internet of Things, Industry 4.0 and smart transport.” The funder had no role in study design, data collection analysis, and interpretation, decision to submit the manuscript for publication, or the preparation and writing of this paper
[35]	Singapore	PubMed, Europe PMC, Scopus, and Web of Science keywords related to ChatGPT and LLMs (“large language model”, “natural language processing”, “generative artificial intelligence”, “ChatGPT”, “chatbot”, “GPT-3.5”, “GPT-4.0”) with those specific to the field of ophthalmology (“ophthalmology”, “ophthalmic”, “ophthalmologist”, “ophthalmological”, “ocular”, “optical”, “eye”, “retina”, “vision science”, “vision research”). published between 1 January 2022 and 31 July 2023	No	32 (24 original research articles and 8 commentaries)	No	ChatGPT 3.5 & 4.0	No	No	National Medical Research Council of Singapore
[24]	Cleveland, USA	Google Scholar, Web of Science, PubMed, and Medline using search terms “ChatGPT” AND “Chatbot” AND “Medical Research” searched on January 21, 2023, at 9:26 PM EST to identify articles published between 2022 and 2023	No	6 (2 literature review articles, 1 case study, 1 editorial, 1 perspective, 1 not specific)	No	Not specified	No	No	No information in the article

^*^RoB = Risk of Bias

**Table 3:** Objectives and key findings of the included systematic reviews and systematic reviews with meta-analysis (n = 17)

Author, Year	Field of study	Objectives related to ChatGPT	Pros of using ChatGPT	Cons/ Challenges of using ChatGPT	Future directions suggested by author (evidence gap)	Findings of Meta—Analysis
[10]	Patient care and medical research	To explore the capacity of ChatGPT in enhancing patient care and its contribution to medical research and medical writing	ChatGPT can help with handling patient inquiries, documenting notes, making decisions, enrolling in trials, managing data, providing decision support, assisting with research, and educating patients	The provided solutions often fall short and present conflicting information, leading to concerns about their originality, privacy, accuracy, bias, and legality. Content generated by ChatGPT raises issues related to bias and potential plagiarism	Longitudinal studies on the role of ChatGPT in healthcare and medical education and research as well as comparison of ChatGPT with other AI tools	N/A
[27]	Support of ChatGPT in conducting a medical systematic review	To evaluate human researchers' performance versus ChatGPT in systematic review tasks (inter- and intrarater reliability, sensitivity, specificity, accuracy, precision, chance hit rate)	A substantial level of agreement between ChatGPT and human researchers in extracting information for systematic review except for reporting study design, clinical task, and clinical implementation	The limitations of ChatGPT render augmented systematic reviews inefficient for experienced researchers. Ethical implications of using ChatGPT in medical scientific writing	Range of application of ChatGPT and other transformer based models in healthcare should be increased	N/A
[25]	Healthcare education, practice and research	To explore the effectiveness of ChatGPT in healthcare Education, research, and practice, while also emphasizing its potential drawbacks	Research: Review and writing, data analysis, code generation, saving Time for tasks requiring human intelligence Practice: drug discovery and development, workflow optimization, cost reduction, documentation, personalized medicine, health literacy Education: personalized learning, critical thinking and problem-based learning	Ethical concerns, copyright issues, transparency challenges, and legal considerations, along with the potential for bias, Plagiarism, lack of originality, inaccurate content leading to hallucination, limited knowledge, incorrect citations, cybersecurity risks, and the threat of infodemics	Potential areas to be explored include the application of AI in reviewing and editing tasks (for journals) and incorporation of Emotional support in patient care involving the use of ChatGPT. Role of ChatGPT in refining communication skills is also an area of importance	N/A
[18]	Treatment decisions (for professionals and public)	To explore the usage and pitfalls of ChatGPT in healthcare	The question-and-answer (QA) design of ChatGPT's interface facilitates integration into the current clinical workflow	Unsuitable for direct clinical deployment as it is not designed for clinical applications	Future development of ‘ChatGPT medical professional version’, specific according to medical specialities	N/A
[36]	Healthcare practice	To investigate the various applications, potential benefits and risks, ethical issues of ChatGPT in healthcare and recommendations for its adoption in medicine and cellular imaging	1. Disseminates critical information about pandemics and infectious diseases 2. virtual assistant for orthognathic surgery consultations 3. addresses inquiries related to dental practice 4. simplifies complex medical concepts 5. differential diagnoses based on patient history 6. analysis of medical images and cellular imaging 7. expedited research processes through quick literature review	1.Inability to understand specialized medical terminology 2. Can augment but not replace human judgment in clinical settings 3. In case of inaccurate clinical advice, accountability (legal and ethical regulatory frameworks) have not yet been devised 4. Balancing automation with the human touch	Work should be done for dynamic and real time learning of ChatGPT for obtaining updated information in medicine and health care	N/A
[31]	Radiology	Evaluating the current applications and future directions of ChatGPT in the field of radiology	Demonstrates substantial potential to augment decision-making and optimizing workflow	Concerns regarding radiologic image processing, ethical and legal implications, potential self-diagnosis and self-management by patients	The image processing potential of ChatGPT 4.0 needs to be validated further	N/A
[2]	Medical and dental research	To evaluate the reliability and accuracy of ChatGPT in medical and dental research	Meta-analysis shows that the accuracy of ChatGPT in providing correct responses was significantly higher compared to the total responses to queries related to medical examination, systematic reviews, clinical reasoning, diagnostic imaging, liver diseases, and COVID-19 vaccination	Transparency, ethical concern, erroneous content, variation in population, data reproducibility	The studies on ChatGPT have small data points tested (small sample size) which poses threat to extrapolation of findings. Therefore, larger datasets (including concepts, queries, prompts etc.) should be tested using ChatGPT to improve the generalizability of the study results	The meta-analysis showed an odds ratio (OR) of 2.25 and a relative risk (RR) of 1.47 with a 95% confidence interval (CI), indicating that the accuracy of ChatGPT in providing correct responses was significantly higher compared to the total responses for queries
[3]	Neurosurgery	To examine the potential benefits and limitations of ChatGPT in neurosurgical practice and education	Personalized treatment plans, supporting surgical planning and navigation, and enhancing large data processing efficiency and accuracy	Question format limitations, validation challenges, and algorithmic bias along with ethical issues related to its usage	Incorporating longitudinal patient data into predictive models can enhance outcome prediction	N/A
[17]	Medical examinations	To assess the performance in medical examinations with multiple-choice questions	ChatGPT correctly answered the majority of multiple-choice questions in medical examinations with a passing grade	Preparations for medical examinations using ChatGPT should be done with caution	Development of training data set specific for medical education Exploring the role of Future AI chatbots in medical examination preparation	Overall performance of ChatGPT ranged from 40% in the biomedical admission test to 100% in a diabetes knowledge questionnaire. The mean performance of ChatGPT was 61.1% (95% CI 56.1%–66.0%
[20]	Medicine and healthcare	To explore the potential of ChatGPT in medicine	Streamline and simplify complex tasks, improve patient care, enhance clinical decision making, and facilitate communication among healthcare professionals	Privacy, ethical considerations, tokenization, sensitivity of wording of prompt, lack of capacity to handle image based questions	Involvement of medical practitioners in training ChatGPT	N/A
[15]	Gastroenterology	To assess the applications, benefits and limitations of ChatGPT in the field of gastroenterology	It can provide recommendations, enhance communication between patients and caregivers, and prompt valuable research inquiries	Obstacles in decoding intricate medical questions, yielded inconsistent responses at times, and exhibited limitations in generating novel content	The model of ChatGPT used in research influences its outcomes, so a comparable version should be used Research on prompt selection for ChatGPT is lacking which can drastically change the outcome/ response	N/A
[32]	Public health dentistry	To find out applications and drawbacks of ChatGPT in public dental health schooling, writing for academic use, research and clinical practice in public dental health	Helps scholars with the authoring of scientific research and dental studies Scientists can focus and allocate more time on experimentation by delegating some tasks to ChatGPT	Prejudice in the training data, undervaluing human skills, possible fraud, legal and reproducibility concerns	Inclusion of more homogenous studies in terms of quality in order to improve the generalizability of systematic review findings	N/A
[22]	Neurosurgery	To explore the potential benefits and limitations of ChatGPT in the field of neurosurgery	Accuracy and efficiency of neurosurgical procedures, as well as diagnosis, treatment, and patient outcomes	1. Need for large datasets 2. Potential for errors in the output	To provide extensive database necessary to train ChatGPT without breaching patient confidentiality	N/A
[4]	Radiology	To conduct a qualitative and quantitative analysis of ChatGPT literature in radiology, assessing its scope and impact	Enhances patient education, protocol selection, and differential diagnosis generation Improves radiology report structuring and examination preparation	1. Inconsistency in performance and information accuracy 2. Challenges in fully integrating AI into clinical radiology workflows	Research on factually incorrect information/ hallucinations generated from ChatGPT is needed	N/A
[5]	Disease risk detection, personalized healthcare procedures, and enhancing patient care safety and quality	To explore the efficacy of ChatGPT and generative AI tools in medical diagnostics, treatment recommendations, and improving healthcare practices	1. Enhances diagnostic accuracy and surgical planning 2. Reduces administrative tasks, improving physician efficiency 3. Supports evidence-based decision-making and clinical education	1. Potential inaccuracies in clinical letter generation 2. Requires regulation and careful integration into clinical workflows 3. Risk of misinterpreting treatment guidelines affecting patient care	Predictive analytics to observe how well can ChatGPT assess the real-time data streams	N/A
[35]	Ophthalmology	To evaluate the effectiveness and potential of ChatGPT in improving ophthalmological care, specifically in diagnosis, patient interaction, and educational roles	1. Offers rapid, accessible information and support for clinical decisions 2. Enhances patient education through simplified explanations of conditions and treatments	1. Potential inaccuracies in medical advice or diagnostic information 2. Ethical concerns around patient data privacy and the reliability of AI-generated advice	Explore the effectiveness of ChatGPT in a diverse linguistic landscape	N/A
[24]	Medical research	To evaluate ChatGPT's application and effectiveness in medical research, including treatment, diagnosis, medication provision, and more	Offers potential benefits in drug development, medical report improvement, providing treatment and medical information, literature review writing, research conduction, data analysis, and personalized medicine	Concerns about ChatGPT's accuracy, originality, academic integrity, and ethical issues like privacy and security in medical research	Overcome issues pertaining to academic integrity, privacy, and ethics	N/A

Table 4 outlines the references of articles reporting each theme and sub theme of ChatGPT within the context of healthcare. According to the included articles, the role of ChatGPT in healthcare from both the patient and caregiver perspectives, emerged as the most frequently studied theme (studied in 16 out of 17 articles) [2–5, 15, 18, 20], Muftić, Kadunić, Mušinbegović, & Almisreb, 2023; [22, 24],Salam, 2023; [27, 31, 32, 35, 36]. The education of patients—in terms of general information gathering about disease—was explored in 11 studies [2–5, 18, 20, 22, 24, 25, 35, 36].

**Table 4:** Major themes and sub themes derived from systematic reviews for the umbrella review evidence synthesis

Themes	Sub-Theme	References (article number) in which this theme was reported*	Frequency of articles reporting the respective theme	Quality of studies assessment
Health services (1)	Diagnosis and clinical decision making (1a)	1, 2, 3, 4, 5, 6, 7, 8, 10, 11, 12, 13, 14, 15, 16, 17	16	
	Treatment options (1b)	1, 3, 4, 5, 8, 11, 12, 13, 15, 16, 17	11	
	Reduce burden on health care professionals (1c)	5, 8, 12, 13, 15	5	
	Health records (1d)	1, 2, 3, 5, 8, 12, 16, 17	8	
	Patient education (1e)	3, 4, 5, 7, 8, 10, 13, 14, 15, 16, 17	11	
Consumers/Patients (2)	Self-diagnosis/management of disease (2a)	5, 11, 12, 15 16, 17	6	
Research (3)	Conducting systematic review (3a)	2, 7	2	
	Research ideas generation (3b)	11	1	
	Collecting and summarising evidence (3c)	1, 2, 3, 6, 7, 12, 17	7	
	Reporting of evidence (scientific writing) (3d)	1 2, 3, 6, 8, 12, 13, 14, 16	9	
	Helps researchers’ direct attention on parts of research requiring intellect (3e)	3, 4, 12, 17	4	
	Data analysis (3f)	17	1	
Medical education (4)	Conducting Assessments (4a)	6, 9, 11, 14, 16,	5	
	Learning (4b)	1, 3, 4, 5, 7, 9, 10	7	
	Integration of AI into curriculum (3c)	12	1	

^*^Article serial number is as per Table 1

Each circle indicates one study Low quality review

Moderate quality review

Additional File 2, Table S1 shows the comparison of various versions of ChatGPT used in the included articles. Eight studies out of 17 mentioned the impact of different versions of ChatGPT on tasks they can perform effectively [2, 4, 15, 17, 18, 27, 31, 35]. ChatGPT 3.5 was found to be less precise and needed human verification but its accuracy depends on the quality of training data. It easily integrates into clinical workflows and is a promising educational tool. ChatGPT 4.0 was able to handle complex tasks such as radiology [31] but was less reliable in less complex tasks [4]. However, gastroenterology self-assessment could not be done by both versions in a satisfactory manner [15].

## Discussion

This umbrella review synthesised 17 existing systematic reviews and meta-analyses investigating the applications, strengths, limitations, and future directions of using ChatGPT in healthcare. The evidence suggests that ChatGPT has diverse applications, which explored enhancing patient care [10], conducting and reporting systematic reviews [27], advancing healthcare education [25], augmenting clinical decision-making [31], and providing preparatory materials for medical examinations [17]. Several studies suggested that ChatGPT can be employed as a valuable tool in clinical practice, assisting clinicians with patient inquiries, writing medical notes and discharge summaries, and making informed decisions about treatment plans. Additionally, it has the potential to serve as a personalised learning tool, encouraging critical thinking and problem-based learning among medical professionals [18, 36].

ChatGPT has demonstrated remarkable capabilities in generating human-like text and conducting natural language processing for text organisation and summarisation. It can expedite processes such as collecting questionnaire responses or conducting interviews, enhancing the effectiveness and efficiency of epidemiological research. Furthermore, ChatGPT supports researchers in locating essential information, developing hypotheses, and analysing data [24]. In healthcare education, ChatGPT serves as a preparatory tool for medical examinations, where it correctly answers most multiple-choice questions, suggesting its potential utility in evaluating medical knowledge [17]. Specialized applications, such as aiding in surgical planning, image recognition, diagnosis, and patient care in neurosurgery [3, 33] and supporting dentistry practices [32], further highlight its transformative potential. However, it is important to note that ChatGPT cannot replace the holistic care provided by a dentist, as decision-making in dentistry is multidisciplinary and involves patient care beyond diagnosis [32].

Administrative efficiency is another domain where ChatGPT shows promise. Its robust linguistic capabilities make it highly suitable for handling intricate administrative tasks, which can significantly aid in busy healthcare settings. Tasks such as managing medical records, generating discharge summaries, formatting examination reports and drafting referral letters are efficiently managed by AI through initial information structuring and organisation. Subsequent review and confirmation by healthcare professionals facilitate the rapid organisation of clinical data, alleviating both time and manpower burdens. This contributes to improving the healthcare environment and the quality of patient care [3, 10, 25, 27, 36].

While AI holds the potential to assume numerous responsibilities currently undertaken by human physicians, such as diagnosis and medication prescription, several limitations must be considered. Studies have raised concerns regarding ChatGPT’s potential for bias, plagiarism, lack of originality, and ethical and legal dilemmas [2, 22, 25]. It frequently produces erroneous or inconsistent content, including inaccurate citations and fabrications, which constrain its reliability in clinical and academic contexts [5, 15]. Furthermore, ChatGPT has difficulty interpreting specialised medical terminology, integrating into clinical workflows and addressing complex medical inquiries [4, 31, 32]. These limitations can lead to a loss of human critical thinking and involvement, as excessive reliance on AI could reduce the exercise of essential cognitive skills, potentially hindering professional growth and societal advancement [30, 36].

In terms of scientific writing, ChatGPT's linguistic capabilities can assist authors in generating ideas, summarising text, editing language, and proofreading documents. However, it is important to note that under the current International Committee of Medical Journal Editors (ICMJE)/Committee on Publication Ethics (COPE) guidelines, ChatGPT is not eligible for authorship in scientific publications unless these guidelines are updated [25]. Moreover, several ethical concerns, including copyright issues, transparency, and the risk of spreading misinformation, have been raised regarding its use in scientific writing [2, 25]. Given these concerns, it is essential to investigate the research domain from the viewpoints of editors, reviewers and journals to develop appropriate policies. Further research is also needed on educational policy formulation and the integration of ChatGPT into teaching methods and curriculum development [37]. Exploring the intersecting realms of research and education offers another avenue for exploration.

While acknowledging the potential significance of ChatGPT in healthcare, the reviewed studies highlight several challenges. ChatGPT’s integration into healthcare systems requires collaboration between AI developers, healthcare professionals and policymakers to maximise its transformative impact. Since the effectiveness of ChatGPT’s outputs depends on the quality and diversity of its training data [6], it is crucial to ensure that it incorporates a broad range of clinical information that accurately reflects the target patient population [31]. This may involve developing specialised ChatGPT models tailored to specific patient groups or healthcare domains to ensure the relevance and efficacy of its outputs.

To ensure responsible deployment, robust validation mechanisms, including expert review and clinical testing are necessary to address issues like AI hallucination, misinformation and bias. In addition, clear privacy regulations and transparent data usage policies are essential to protect user data and build trust in AI-generated responses. Establishing ethical frameworks, certification standards, and promoting digital literacy through educational initiatives will empower users to understand ChatGPT’s limitations and use it responsibly [13, 38].

With patients increasingly gaining access to ChatGPT, concerns may arise regarding self-diagnosis and the potential for cyberchondria [29]. While empowering patients with information can enhance autonomy and engagement in their healthcare, it also raises concerns about the accuracy and interpretation of medical data. Self-diagnosis based solely on ChatGPT’s outputs could lead to misinterpretation or oversight of critical details, potentially compromising patient safety. Therefore, it is crucial to establish guidelines and educational resources to support patients in using ChatGPT as a supplementary tool rather than a substitute for professional medical advice and diagnosis.

This umbrella review demonstrates both strengths and limitations of ChatGPT. We conducted it by relying on existing systematic reviews and meta-analyses, ensuring methodological rigour through adherence to PRISMA guidelines and the use of the AMSTAR-2 tool for quality assessment. Our stringent criteria for study inclusion aimed to analyse high-quality, relevant research, while meticulous search strategies and transparent selection criteria minimised biases. Despite efforts to standardise methodologies and terminologies, integrating and reconciling inconsistencies across studies posed challenges. While our review provided a comprehensive overview, it lacked detailed insights into specific healthcare contexts, emphasising the need for further primary research. Moreover, Generative AI is a dynamic field that undergoes regular updates, making comparisons between different versions of ChatGPT valuable for future researchers important. Newer versions generally demonstrate enhanced natural language processing capabilities, which can significantly benefit healthcare applications. However, concerns regarding the reliability of newer versions, such as ChatGPT 4.0, which performed poorly in handling simpler queries compared to its predecessor, highlight the need for further investigation into these advancements (Additional File 2). Addressing these limitations will enhance the robustness and applicability of our findings for evidence-based decision-making in healthcare practice. Moreover, longitudinal studies are necessary to examine the broader, long-term impact of ChatGPT on healthcare systems, patient outcomes, workflow efficiency, and provider-patient dynamics. Combining these approaches will ensure a holistic understanding of ChatGPT’s role in advancing healthcare while addressing its limitations.

## Conclusions

The ChatGPT’s integration into healthcare as a reliable educational, research and clinical augmentation tool shows immense promise however, its success relies on the establishment of robust regulations and control mechanisms to ensure ethical deployment.

ChatGPT’s version 3.5 was found to be more reliable in certain circumstances while complex tasks can be handled well by the ChatGPT version 4.0. Prioritising ethical considerations is essential to harness AI's potential while preserving trust and integrity in healthcare and research practices. Acknowledging and addressing challenges such as ethical concerns, bias and the potential for overreliance is crucial.

Through collaborative efforts among stakeholders, ChatGPT can significantly enhance healthcare delivery, research innovation and patient outcomes, marking a step forward in ethically responsible use of AI in the healthcare field.

## Supplementary Information

Additional file 1.

Additional file 2.

## Data Availability

Not applicable.

## Abbreviations

AMSTAR-2: A MeaSurement Tool to Assess systematic Reviews
ChatGPT: Chat Generative Pre-trained Transformer
SR: Systematic reviews
SRMA: Systematic reviews with meta-analysis
ICMJE: International Committee of Medical Journal Editors
COPE: Committee on Publication Ethics
PRISMA: Preferred Reporting Items for Systematic Reviews and Meta-Analysis

## References

[1] Ashraf H, Ashfaq H. The role of ChatGPT in medical research: progress and limitations. Ann Biomed Eng. 2023. 10.1007/s10439-023-03311-0. 37452215 10.1007/s10439-023-03311-0

[2] Bagde H, Dhopte A, Alam MK, Basri R. A systematic review and meta-analysis on ChatGPT and its utilization in medical and dental research. Heliyon. 2023;9(12): e23050. 10.1016/j.heliyon.2023.e23050. 38144348 10.1016/j.heliyon.2023.e23050 PMC10746423

[3] Bečulić H, Begagić E, Skomorac R, Mašović A, Selimović E, Pojskić M. 2024. ChatGPT’s contributions to the evolution of neurosurgical practice and education: A systematic review of benefits, concerns and limitations. Medicinski Glasnik Ljekarske Komore Zenicko-Dobojskog Kantona. 10.17392/1661-23 10.17392/1661-23 37950660

[4] Bera K, O’Connor G, Jiang S, Tirumani SH, Ramaiya N. Analysis of ChatGPT publications in radiology: literature so far. Curr Probl Diagn Radiol. 2024;53(2):215–25. 10.1067/j.cpradiol.2023.10.013. 37891083 10.1067/j.cpradiol.2023.10.013

[5] Bugaj M, Kliestik T, Lăzăroiu G. Generative artificial intelligence-based diagnostic algorithms in disease risk detection, in personalized and targeted healthcare procedures, and in patient care safety and quality. Contemp Read Law Soc Justice. 2023;15(1):9–26.

[6] Cao Y, Li S, Liu Y, Yan Z, Dai Y, Yu PS, Sun L. 2023. A comprehensive survey of AI-generated content (AIGC): a history of generative AI from GAN to ChatGPT (arXiv:2303.04226). arXiv. http://arxiv.org/abs/2303.04226

[7] Cazzato G, Capuzzolo M, Parente P, Arezzo F, Loizzi V, Macorano E, Marzullo A, Cormio G, Ingravallo G. Chat GPT in diagnostic human pathology: will it be useful to pathologists? A preliminary review with ‘query session’ and future perspectives. AI. 2023;4(4):1010–22. 10.3390/ai4040051.

[8] Choi GJ, Kang H. Introduction to umbrella reviews as a useful evidence-based practice. J Lipid Atherosclerosis. 2023;12(1):3. 10.12997/jla.2023.12.1.3 PMC9884555 36761061

[9] Gabashvili I. 2023. ChatGPT in dermatology: a comprehensive systematic review. medRxiv, 2023–06.

[10] Garg RK, Urs VL, Agarwal AA, Chaudhary SK, Paliwal V, Kar SK. Exploring the role of ChatGPT in patient care (diagnosis and treatment) and medical research: a systematic review. Health Promot Perspect. 2023;13(3):183. 37808939 10.34172/hpp.2023.22 PMC10558973

[11] Haddaway NR, Collins AM, Coughlin D, Kirk S. The role of Google Scholar in evidence reviews and its applicability to grey literature searching. PLoS ONE. 2015;10(9): e0138237. 26379270 10.1371/journal.pone.0138237 PMC4574933

[12] Harry A. The future of medicine: harnessing the power of AI for revolutionizing healthcare. Int J Multidis Sci Arts. 2023;2(1):36–47.

[13] Hastings J. Preventing harm from non-conscious bias in medical generative AI. Lancet Digit Health. 2024;6(1):e2–3. 10.1016/S2589-7500(23)00246-7[publishedOnlineFirst:2023/12/21]. 38123253 10.1016/S2589-7500(23)00246-7

[14] Javaid M, Haleem A, Singh RP. ChatGPT for healthcare services: an emerging stage for an innovative perspective. BenchCouncil Trans Benchmarks Standards Evaluat. 2023;3(1): 100105.

[15] Klang E, Sourosh A, Nadkarni GN, Sharif K, Lahat A. Evaluating the role of ChatGPT in gastroenterology: a comprehensive systematic review of applications, benefits, and limitations. Ther Adv Gastroenterol. 2023;16:17562848231218618. 10.1177/17562848231218618. 10.1177/17562848231218618 PMC10750546 38149123

[16] Lee D, Yoon SN. Application of artificial intelligence-based technologies in the healthcare industry: opportunities and challenges. Int J Environ Res Public Health. 2021;18(1):271. 33401373 10.3390/ijerph18010271 PMC7795119

[17] Levin G, Horesh N, Brezinov Y, Meyer R. Performance of ChatGPT in medical examinations: a systematic review and a meta-analysis. Int J Obstet Gynaecol. 2024;131(3):378–80. 10.1111/1471-0528.17641. 10.1111/1471-0528.17641 37604703

[18] Li J, Dada A, Puladi B, Kleesiek J, Egger J. ChatGPT in healthcare: a taxonomy and systematic review. Comput Methods Programs Biomed. 2024;245: 108013. 10.1016/j.cmpb.2024.108013. 38262126 10.1016/j.cmpb.2024.108013

[19] Mitsea E, Drigas A, Skianis C. Digitally assisted mindfulness in training self-regulation skills for sustainable mental health: a systematic review. Behav Sci. 2023;13(12):1008. 38131865 10.3390/bs13121008 PMC10740653

[20] Muftić F, Kadunić M, Mušinbegović A, Abd Almisreb A. Exploring medical breakthroughs: a systematic review of ChatGPT applications in healthcare. Southeast Eur J Soft Comput. 2023;12(1):13–41.

[21] Rao A, Kim J, Kamineni M, Pang M, Lie W, Succi MD. Evaluating ChatGPT as an adjunct for radiologic decision-making. medRxiv. 2023;16:2023–02. 10.1016/j.jacr.2023.05.003 PMC10733745 37356806

[22] Roman A, Al-Sharif L, Al Gharyani M. The expanding role of ChatGPT (chat-generative pre-trained transformer) in neurosurgery: a systematic review of literature and conceptual framework. Cureus. 2023. 10.7759/cureus.43502. 37719492 10.7759/cureus.43502 PMC10500385

[23] Roumeliotis KI, Tselikas ND. ChatGPT and open-AI models: a preliminary review. Future Internet. 2023;15(6):192.

[24] Ruksakulpiwat S, Kumar A, Ajibade A. Using ChatGPT in medical research: current status and future directions. J Multidiscip Healthc. 2023;16:1513–20. 10.2147/JMDH.S413470. 37274428 10.2147/JMDH.S413470 PMC10239248

[25] Sallam M. ChatGPT utility in healthcare education, research, and practice: systematic review on the promising perspectives and valid concerns. Healthcare. 2023;11(6):887. 10.3390/healthcare11060887. 36981544 10.3390/healthcare11060887 PMC10048148

[26] Sarkis-Onofre R, Catalá-López F, Aromataris E, Lockwood C. How to properly use the PRISMA statement. Syst Rev. 2021;10(1):117. 10.1186/s13643-021-01671-z. 33875004 10.1186/s13643-021-01671-z PMC8056687

[27] Schopow N, Osterhoff G, Baur D. Applications of the natural language processing tool ChatGPT in clinical practice: comparative study and augmented systematic review. JMIR Med Inform. 2023;11: e48933. 38015610 10.2196/48933 PMC10716749

[28] Shea BJ, Reeves BC, Wells G, Thuku M, Hamel C, Moran J, Moher D, Tugwell P, Welch V, Kristjansson E. AMSTAR 2: a critical appraisal tool for systematic reviews that include randomised or non-randomised studies of healthcare interventions, or both. Bmj. 2017. 10.1136/bmj.j4008. 28935701 10.1136/bmj.j4008 PMC5833365

[29] Starcevic V, Berle D, Arnáez S. Recent insights into cyberchondria. Curr Psychiatry Rep. 2020;22(11):56. 10.1007/s11920-020-01179-8. 32852626 10.1007/s11920-020-01179-8 PMC7450158

[30] Swaminathan A, Rathnasabapathy M. Role of creativity in problem solving–a review. Rev Int Geograph Educ Online. 2021;11(8):2.

[31] Temperley HC, O’Sullivan NJ, Mac Curtain BM, Corr A, Meaney JF, Kelly ME, Brennan I. Current applications and future potential of C hat GPT in radiology: a systematic review. J Med Imaging Radiat Oncol. 2024;1754–9485:13621. 10.1111/1754-9485.13621. 10.1111/1754-9485.13621 38243605

[32] Tiwari A, Kumar A, Jain S, Dhull KS, Sajjanar A, Puthenkandathil R, Paiwal K, Singh R. Implications of ChatGPT in public health dentistry: a systematic review. Cureus. 2023. 10.7759/cureus.40367. 37456464 10.7759/cureus.40367 PMC10340128

[33] Iqbal U, Lee LT, Rahmanti AR, Celi LA, Li YJ. Can large language models provide secondary reliable opinion on treatment options for dermatological diseases? J Am Med Inform Assoc. 2024;31(6):1341–7. 10.1093/jamia/ocae067.PMID:38578616;PMCID:PMC11105123. 38578616 10.1093/jamia/ocae067 PMC11105123

[34] Wang C, Liu S, Yang H, Guo J, Wu Y, Liu J. Ethical considerations of using ChatGPT in health care. J Med Int Res. 2023;25: e48009. 10.2196/48009 PMC10457697 37566454

[35] Wong M, Lim ZW, Pushpanathan K, Cheung CY, Wang YX, Chen D, Tham YC. Review of emerging trends and projection of future developments in large language models research in ophthalmology. Br J Ophthalmol. 2023. 10.1136/bjo-2023-324734. 38164563 10.1136/bjo-2023-324734

[36] Younis HA, Eisa TAE, Nasser M, Sahib TM, Noor AA, Alyasiri OM, Salisu S, Hayder IM, Younis HA. A systematic review and meta-analysis of artificial intelligence tools in medicine and healthcare: applications, considerations, limitations. Motiv Challenges Diagn. 2024;14(1):109. 10.3390/diagnostics14010109. 10.3390/diagnostics14010109 PMC10802884 38201418

[37] Yu H. The application and challenges of ChatGPT in educational transformation: new demands for teachers’ roles. Heliyon. 2024;10(2): e24289. 10.1016/j.heliyon.2024.e24289. 38298626 10.1016/j.heliyon.2024.e24289 PMC10828640

[38] Zack T, Lehman E, Suzgun M, et al. Assessing the potential of GPT-4 to perpetuate racial and gender biases in health care: a model evaluation study. Lancet Digit Health. 2024;6(1):e12–22. 10.1016/S2589-7500(23)00225-X[publishedOnlineFirst:2023/12/21]. 38123252 10.1016/S2589-7500(23)00225-X

