# Supplementary material for: Impact of large language model (ChatGPT) in healthcare: an umbrella review and evidence synthesis
Source: J Biomed Sci. 2025 May 7;32:45. doi: 10.1186/s12929-025-01131-z (PMC12057020; doi:10.1186/s12929-025-01131-z)
Supplement: Supplementary file 1 — Additional file 1. [file 12929_2025_1131_MOESM1_ESM.docx]

**Supplementary Information**

**Title: Impact of ChatGPT in Healthcare: An Umbrella Review and Evidence Synthesis**

**Additional File 1: Specific search query for Google Scholar, PubMed, Scopus, and Web of Science**

**Search query for Google Scholar (using “keywords and Boolean operators”)**

[ "Systematic Review" AND "ChatGPT" AND "Consumer Health Informatics" OR "Consumer Health Information" OR "Patient education" OR "Health information seeking" OR mHealth OR eHealth OR Telehealth OR "Digital health intervention*" OR "Health literacy" OR "Health communication" OR "Health technology" OR "Patient empowerment" OR "Health behaviour" OR "Health promotion" OR "Health education" OR "Health informatics" OR "Mobile health" OR "Electronic health" OR "Telemedicine" OR "Health technology adoption" OR "Patient Education as Topic" OR "Health Information Seeking Behaviour" OR "Mobile Health Units" OR "Telemedicine" OR "Health Literacy" OR "Health Communication" OR "Health Promotion" OR "Health Education" OR "Health Behavior" OR "Health Technology Assessment" OR "Patient Empowerment" OR "Medical Informatics" OR "Health Knowledge, Attitudes, Practice" OR "Health Services Accessibility" OR "Health Information Systems" OR "Diagnosis" OR "Diagnostic" OR "Diagnose" OR "Diagnosis and prevention" OR "Prevent" OR "Prevention" OR "Prevention Strategy*" OR "Disease identification" OR "Disease prediction" OR "Patient self-diagnosis" OR "Health risk assessment" OR "Preventative healthcare" OR "Health screening" OR "Risk assessment" OR "Disease prevention" OR "Early detection" OR "Healthcare prevention" OR "Preventive medicine" OR "Prophylaxis" OR "Health promotion" OR "Disease Identification" OR "Disease Prediction" OR "Patient Self-Diagnosis" OR "Health Risk Assessment" OR "Preventive Healthcare" OR "Mass Screening" OR "Disease Prevention" OR "Early Detection of Disease" OR "Health Promotion" OR "Preventive Medicine" OR "Prophylaxis"].

**Search query for PubMed (using “title/abstract”)**

(("Systematic Review"[Title/Abstract] OR "Meta-Analysis"[Title/Abstract] OR "Systematic Reviews as Topic"[Mesh] OR "Meta-Analysis as Topic"[Mesh]) AND "ChatGPT"[Title/Abstract]) AND ("Consumer Health Informatics"[Title/Abstract] OR "Consumer Health Information"[Title/Abstract] OR "Patient education"[Title/Abstract] OR "Health information seeking"[Title/Abstract] OR mHealth[Title/Abstract] OR eHealth[Title/Abstract] OR Telehealth[Title/Abstract] OR "Digital health intervention*"[Title/Abstract] OR "Health literacy"[Title/Abstract] OR "Health communication"[Title/Abstract] OR "Health technology"[Title/Abstract] OR "Patient empowerment"[Title/Abstract] OR "Health behaviour"[Title/Abstract] OR "Health promotion"[Title/Abstract] OR "Health education"[Title/Abstract] OR "Health informatics"[Title/Abstract] OR "Mobile health"[Title/Abstract] OR "Electronic health"[Title/Abstract] OR "Telemedicine"[Title/Abstract] OR "Health technology adoption"[Title/Abstract] OR "Patient Education as Topic"[Mesh] OR "Health Information Seeking Behaviour"[Mesh] OR "Mobile Health Units"[Mesh] OR "Telemedicine"[Mesh] OR "Health Literacy"[Mesh] OR "Health Communication"[Mesh] OR "Health Promotion"[Mesh] OR "Health Education"[Mesh] OR "Health Behavior"[Mesh] OR "Health Technology Assessment"[Mesh] OR "Patient Empowerment"[Mesh] OR "Medical Informatics"[Mesh] OR "Health Knowledge, Attitudes, Practice"[Mesh] OR "Health Services Accessibility"[Mesh] OR "Health Information Systems"[Mesh] OR "Diagnosis"[Mesh] OR "Diagnostic"[Title/Abstract] OR "Diagnose"[Title/Abstract] OR "Diagnosis and prevention"[Title/Abstract] OR "Prevent"[Title/Abstract] OR "Prevention"[Title/Abstract] OR "Prevention Strategy*"[Title/Abstract] OR "Disease identification"[Title/Abstract] OR "Disease prediction"[Title/Abstract] OR "Patient self-diagnosis"[Title/Abstract] OR "Health risk assessment"[Title/Abstract] OR "Preventative healthcare"[Title/Abstract] OR "Health screening"[Title/Abstract] OR "Risk assessment"[Title/Abstract] OR "Disease prevention"[Title/Abstract] OR "Early detection"[Title/Abstract] OR "Healthcare prevention"[Title/Abstract] OR "Preventive medicine"[Title/Abstract] OR "Prophylaxis"[Title/Abstract] OR "Health promotion"[Title/Abstract] OR "Disease Identification"[Title/Abstract] OR "Disease Prediction"[Title/Abstract] OR "Patient Self-Diagnosis"[Title/Abstract] OR "Health Risk Assessment"[Title/Abstract] OR "Preventive Healthcare"[Title/Abstract] OR "Mass Screening"[Title/Abstract] OR "Disease Prevention"[Title/Abstract] OR "Early Detection of Disease"[Title/Abstract] OR "Health Promotion"[Title/Abstract] OR "Preventive Medicine"[Title/Abstract] OR "Prophylaxis"[Title/Abstract]) NOT "Scoping Review"[Title/Abstract]

**Search query for Scopus (using “title/abstract/keywords”)**

TITLE-ABS-KEY (("Systematic Review" AND "ChatGPT") AND ("Consumer Health Informatics" OR "Consumer Health Information" OR "Patient education" OR "Health information seeking" OR mHealth OR eHealth OR Telehealth OR "Digital health intervention*" OR "Health literacy" OR "Health communication" OR "Health technology" OR "Patient empowerment" OR "Health behaviour" OR "Health promotion" OR "Health education" OR "Health informatics" OR "Mobile health" OR "Electronic health" OR Telemedicine OR "Health technology adoption" OR "Patient Education as Topic" OR "Health Information Seeking Behaviour" OR "Mobile Health Units" OR "Telemedicine" OR "Health Literacy" OR "Health Communication" OR "Health Promotion" OR "Health Education" OR "Health Behavior" OR "Health Technology Assessment" OR "Patient Empowerment" OR "Medical Informatics" OR "Health Knowledge, Attitudes, Practice" OR "Health Services Accessibility" OR "Health Information Systems" OR "Diagnosis" OR "Diagnostic" OR "Diagnose" OR "Diagnosis and prevention" OR "Prevent" OR "Prevention" OR "Prevention Strategy*" OR "Disease identification" OR "Disease prediction" OR "Patient self-diagnosis" OR "Health risk assessment" OR "Preventative healthcare" OR "Health screening" OR "Risk assessment" OR "Disease prevention" OR "Early detection" OR "Healthcare prevention" OR "Preventive medicine" OR "Prophylaxis"))

**Search query for Web of Science (using “title/topic”)**

TI=(("Systematic Review" AND "ChatGPT") AND ("Consumer Health Informatics" OR "Consumer Health Information" OR "Patient education" OR "Health information seeking" OR mHealth OR eHealth OR Telehealth OR "Digital health intervention*" OR "Health literacy" OR "Health communication" OR "Health technology" OR "Patient empowerment" OR "Health behaviour" OR "Health promotion" OR "Health education" OR "Health informatics" OR "Mobile health" OR "Electronic health" OR Telemedicine OR "Health technology adoption" OR "Patient Education as Topic" OR "Health Information Seeking Behaviour" OR "Mobile Health Units" OR "Telemedicine" OR "Health Literacy" OR "Health Communication" OR "Health Promotion" OR "Health Education" OR "Health Behavior" OR "Health Technology Assessment" OR "Patient Empowerment" OR "Medical Informatics" OR "Health Knowledge, Attitudes, Practice" OR "Health Services Accessibility" OR "Health Information Systems" OR "Diagnosis" OR "Diagnostic" OR "Diagnose" OR "Diagnosis and prevention" OR "Prevent" OR "Prevention" OR "Prevention Strategy*" OR "Disease identification" OR "Disease prediction" OR "Patient self-diagnosis" OR "Health risk assessment" OR "Preventative healthcare" OR "Health screening" OR "Risk assessment" OR "Disease prevention" OR "Early detection" OR "Healthcare prevention" OR "Preventive medicine" OR "Prophylaxis" OR "Health promotion" OR "Disease Identification" OR "Disease Prediction" OR "Patient Self-Diagnosis" OR "Health Risk Assessment" OR "Preventive Healthcare" OR "Mass Screening" OR "Disease Prevention" OR "Early Detection of Disease" OR "Health Promotion" OR "Preventive Medicine" OR "Prophylaxis"))

OR

TS=(("Systematic Review" AND "ChatGPT") AND ("Consumer Health Informatics" OR "Consumer Health Information" OR "Patient education" OR "Health information seeking" OR mHealth OR eHealth OR Telehealth OR "Digital health intervention*" OR "Health literacy" OR "Health communication" OR "Health technology" OR "Patient empowerment" OR "Health behaviour" OR "Health promotion" OR "Health education" OR "Health informatics" OR "Mobile health" OR "Electronic health" OR Telemedicine OR "Health technology adoption" OR "Patient Education as Topic" OR "Health Information Seeking Behaviour" OR "Mobile Health Units" OR "Telemedicine" OR "Health Literacy" OR "Health Communication" OR "Health Promotion" OR "Health Education" OR "Health Behavior" OR "Health Technology Assessment" OR "Patient Empowerment" OR "Medical Informatics" OR "Health Knowledge, Attitudes, Practice" OR "Health Services Accessibility" OR "Health Information Systems" OR "Diagnosis" OR "Diagnostic" OR "Diagnose" OR "Diagnosis and prevention" OR "Prevent" OR "Prevention" OR "Prevention Strategy*" OR "Disease identification" OR "Disease prediction" OR "Patient self-diagnosis" OR "Health risk assessment" OR "Preventative healthcare" OR "Health screening" OR "Risk assessment" OR "Disease prevention" OR "Early detection" OR "Healthcare prevention" OR "Preventive medicine" OR "Prophylaxis" OR "Health promotion" OR "Disease Identification" OR "Disease Prediction" OR "Patient Self-Diagnosis" OR "Health Risk Assessment" OR "Preventive Healthcare" OR "Mass Screening" OR "Disease Prevention" OR "Early Detection of Disease" OR "Health Promotion" OR "Preventive Medicine" OR "Prophylaxis"))
