# Supplementary material for: Impact of large language model (ChatGPT) in healthcare: an umbrella review and evidence synthesis
Source: J Biomed Sci. 2025 May 7;32:45. doi: 10.1186/s12929-025-01131-z (PMC12057020; doi:10.1186/s12929-025-01131-z)
Supplement: Supplementary file 2 — Additional file 2. [file 12929_2025_1131_MOESM2_ESM.docx]

**Supplementary Information**

**Title: Impact of ChatGPT in Healthcare: An Umbrella Review and Evidence Synthesis**

**Additional File 2: Table S1. Comparison of the Impact of ChatGPT Versions on Study Findings**

| **Author, year** | **Version of ChatGPT studied** | **Field of study** | **Study Focus** | **Impact of Version** |
| --- | --- | --- | --- | --- |
| (Schopow et al., 2023) | 3.5 (Legacy) and 4.0 | Support of ChatGPT in conducting a medical systematic review | GPT 3.5 for generating search strategies and criteria, also title and abstract Screening  GPT 4.0 for Summarization and Writing | - GPT 3.5 are good for generating search strategies and defining criteria; less precise in screening tasks. Also effective in screening titles and abstracts but includes more irrelevant results - GPT 4.0 useful for language correction and text modification but limited in complex tasks and less suitable for experienced researchers |
| (Li et al., 2024) | Version released in Nov., 2022 (ChatGPT 3.5) | Treatment decisions (for professionals and public) | Clinical Workflow Integration, Answer and Justification, and Limitation | - This version is easily integrates into clinical workflows, providing feedback in real-time and sometimes contain novel insights and perspectives  - Limited to text input and output. Its justifications are based on probabilistic predictions rather than expert reasoning, which can lead to misleading or nonsensical explanations. The accuracy of its answers depends heavily on the quality of its training data, posing risks of providing biased or dangerous advice |
| (Temperley et al., 2024) | version 3.0 to 4.0 | Radiology | Radiology Applications (generating academic radiology articles, interventional radiology procedures, US board-style examination questions, imaging interpretation) | While GPT-3.5 is useful for certain tasks, it often requires human verification due to its tendency for data inaccuracies and incorrect information. GPT-4, on the other hand, offers enhanced capabilities for handling more complex tasks, particularly in imaging, and shows significant improvement over GPT-3.5. |
| (Bagde et al., 2023) | 3.5 | Medical and dental research | Evaluated the accuracy of ChatGPT in answering medical queries related to medical and dental domains. | ChatGPT demonstrated a wide range of accuracy across various datasets and specialties. Meta-analysis showed significantly higher accuracy in providing correct responses. However, significant heterogeneity suggests variability in effect sizes. While promising, the accuracy of ChatGPT’s responses is not consistently reliable, indicating the need for further research to explore heterogeneity sources and optimize performance. |
| (Levin et al., 2024) | 3.5 | Medical examinations | Medical Examinations (Multiple-Choice Questions) | GPT-3.5 demonstrated good performance on multiple-choice questions, achieving approximately a passing grade. The correct response rate ranged from 40% in biomedical admission tests to 100% in diabetes knowledge questionnaires, with a mean performance of 61.1% (95% CI 56.1%–66.0%). This indicates promise as an educational tool, though improvements in accuracy and consistency are needed. |
| (Klang et al., 2023) | Heterogenous | Gastroenterology | Various applications in Gastroenterology (patient communication, medical education, disease management) | ChatGPT-3.5 and GPT-4 both failed to pass gastroenterology self-assessment tests, scoring 65.1% and 62.4% respectively (passing grade 70%). No significant difference in performance between versions. While promising for tasks like GERD management and guideline adherence, GPT-3.5 struggled with patient education and consistency in self-assessment contexts. |
| (Bera et al., 2023) | ChatGPT version 3, 3.5 and 4 | Radiology | Radiology Applications (comparative performance, clinical tasks, radiology reporting) | GPT-4 generally outperforms GPT-3.5 in higher-order complex questions but may be less reliable in lower-order questions. GPT-3.5 provides more consistent answers in simpler tasks. Both versions can assist in clinical tasks such as differential diagnosis and exam suggestions. GPT-4 shows promise in structuring radiology reports but requires human oversight for accuracy and consistency. |
| (Wong et al., 2023) | ChatGPT 3.5 & 4.0 | Ophthalmology | evaluating ChatGPT’s performance in ophthalmology examinations or clinical tasks | GPT-4 outperforms GPT-3.5 in ophthalmology exams and general medical tasks, such as constructing discharge notes and evaluating diagnoses. Both versions struggled with generating scientific articles or abstracts and had difficulty with specific subdomain questions, particularly those involving specialized treatment options. |
